# Hysterectomy with Bilateral Salpingo-Oophorectomy in Female-to-Male Gender Affirmation Surgery: Comparison of Two Methods

**DOI:** 10.1155/2018/3472471

**Published:** 2018-05-08

**Authors:** M. Jeftovic, B. Stojanovic, M. Bizic, D. Stanojevic, J. Kisic, M. Bencic, M. L. Djordjevic

**Affiliations:** ^1^Belgrade Center for Genital Reconstructive Surgery, Belgrade, Serbia; ^2^School of Medicine, University of Belgrade, Belgrade, Serbia; ^3^University Children's Hospital, Belgrade, Serbia

## Abstract

**Introduction:**

The optimal route for hysterectomy with bilateral salpingo-oophorectomy in female-to-male gender affirmation surgery is still under debate, due to the quite limited and inconsistent published data. The aim of this study is to present and compare the results of vaginal and laparoscopic hysterectomy as part of gender affirmation surgery in female-to-male transsexuals.

**Materials and Methods:**

Between 2012 and 2017, 124 female-to-male transsexuals, aged 18–43 years (mean age: 28.5), underwent hysterectomy with bilateral salpingo-oophorectomy, followed by colpocleisis and gender affirmation surgery. Transvaginal and laparoscopic hysterectomy were performed in 92 and 32 patients, respectively. Standard outcome measures (types and rates of complications, operative time, blood loss, and postoperative hospital stay) were used to compare the two groups of patients.

**Results:**

The mean follow-up was 41 months (ranged from 6 to 65 months). The duration of transvaginal approach was significantly shorter (51 minutes compared to 76 minutes, *p* < 0.001). The total complication rates (less than 3%), reoperation rates (0%), blood loss, and postoperative hospital stays (4.3 days compared to 4.5 days) showed no statistical difference.

**Conclusions:**

Both approaches are safe, with minimal complications. However, we prefer transvaginal hysterectomy due to its shorter operative time, cost-effectiveness, and simpler continuation with one-stage female-to-male gender affirmation surgery.

## 1. Introduction

The goal of female-to-male gender affirmation surgery is to remove all female attributes, accomplish male appearance of the entire body, and ultimately improve the quality of life. Several complex procedures are included: removal of female genitalia (hysterectomy with bilateral salpingo-oophorectomy and vaginectomy), chest masculinization (bilateral mastectomy), and genital reconstructive surgery (metoidioplasty or phalloplasty, urethral lengthening, and scrotoplasty with testicular implants). In many countries, hysterectomy with salpingo-oophorectomy is a condition for legal recognition of the male sex; in addition, all transsexuals consider it extremely important for their well-being. Therefore, it is usually the first surgical step in their transition, and it can be performed as an isolated procedure, or at the same time with mastectomy and/or sex reassignment surgery. Generally, hysterectomy with bilateral salpingo-oophorectomy should be performed as a safe, minimally invasive, and expeditious procedure, with a low risk of complications and fast postoperative recovery. This way, further reconstruction of new male genitalia should not be compromised [1–3].

Although all available hysterectomy procedures have been utilized and reported in transsexuals (abdominal, vaginal, laparoscopic, and robotic-assisted), there is no clear gold standard. Transvaginal hysterectomy, with its numerous advantages, has been our method of choice in female-to-male gender affirmation surgery for decades. We have recently introduced the laparoscopic approach as a possible alternative in selected cases. The aim of our study is to compare transvaginal and laparoscopic hysterectomy with salpingo-oophorectomy as part of gender affirmation surgery, as well as the advantages and disadvantages of the two procedures.

## 2. Materials and Methods

Between January 2012 and March 2017, a total of 124 female transsexuals, aged 18–43 years (mean age: 28.5), underwent hysterectomy with bilateral salpingo-oophorectomy, followed by colpocleisis and gender affirmation surgery (metoidioplasty or total phalloplasty, urethral lengthening, and scrotoplasty). Bilateral mastectomy was performed at the same stage in 42 patients. Single-stage gender affirmation surgery is performed by a team of gynecology specialists and gender surgeons. The approach begins with hysterectomy and proceeds with metoidioplasty or total phalloplasty at the same stage. When bilateral mastectomy is planned, it is performed at the same time as transvaginal hysterectomy or, if laparoscopic hysterectomy is used, simultaneously with metoidioplasty. Indications for laparoscopic approach include patients' preference and nonobese persons. After complete preoperative gynecological evaluation, transvaginal and laparoscopic hysterectomy were performed in 92 and 32 patients, respectively.

All patients were required to fulfill requirements according to WPATH Standards of Care prior to surgery [4]. They had been receiving hormonal treatment for a mean period of 3 years (range: 18 months to 23 years). Testosterone treatment was discontinued two weeks before surgery in all patients.

Transvaginal hysterectomy was performed with bilateral oophorectomy, in a standard manner [5, 6]. After uterine vessels are identified on each side and clamped, uterine fundus is delivered posteriorly to identify, cut, and suture-ligate uteroovarian ligaments. Infundibulopelvic ligament is clamped and suture-tied, and adnexa are removed. Laparoscopic hysterectomy is performed using an optic camera and three trocars. After division of the uterus and cervix from the upper vagina, the uterus and both adnexa are removed [7]. Subsequent vaginectomy is performed by total removal of the vaginal mucosa (colpocleisis), with preservation of the part of the anterior vaginal wall near the urethra, which is used for urethral lengthening [1, 3]. Metoidioplasty and phalloplasty are performed as already described [8, 9]. The clitoris is maximally lengthened by division of all ligaments, dorsally, and straightened by dissection and division of the short urethral plate, ventrally. Phalloplasty is performed using musculocutaneous latissimus dorsi free flap. Neophallus is fixed in the pubic region, and microvascular anastomosis is performed with the blood vessels at the recipient site. In both procedures, urethral lengthening is performed using all available well-vascularized genital flaps combined with buccal mucosa grafts. Silicone testicular implants are inserted into the newly created scrotal pockets. Suprapubic urinary drainage is introduced and left indwelling for three weeks. In case of simultaneous chest masculinization, transareolar approach with reduction of the nipple-areola complex is preferred. In case of large breasts and poor skin elasticity, radical mastectomy is performed, with free grafting of the nipple-areola complex.

There are two patient groups, classified according to the type of hysterectomy with bilateral salpingo-oophorectomy (transvaginal and laparoscopic); the results are compared using standard outcome measures (types and rates of complications, operative time, blood loss, and postoperative hospital stay). Nonparametric Mann–Whitney *U* test and Spearman's rank correlation are used for statistical analysis, with *p* < 0.05 presenting statistically significant result.

## 3. Results

Follow-up ranged from 6 months to 65 months (mean: 41 months). Two patients who underwent transvaginal hysterectomy had previous delivery, and both were uniparous. All other patients were nulliparous. None of the patients had significant gynecological complaints during the preoperative evaluation (Table 1).

Outcomes for the two groups are presented in Table 2. Transvaginal hysterectomy with bilateral salpingo-oophorectomy had shorter operative times (mean: 51 minutes) compared to laparoscopic approach (mean: 76 minutes), with the difference being statistically significant (*p* < 0.05). The type of hysterectomy procedure was probably the predictor of the total operative time for one-stage gender affirmation surgery. Mean duration of the total gender affirmation surgery was shorter in case of transvaginal approach than in the laparoscopic approach (Table 3). Postoperative hospital stay ranged from 3 to 8 days, depending on the type of sex reassignment surgery (metoidioplasty or total phalloplasty). Mean hospital stay in transvaginal and laparoscopic group was 4.3 and 4.5 days, respectively, and the difference was not statistically significant (*p* = 0.897). No correlation between operative time and postoperative hospital stay was observed for transvaginal (*p* = 0.162) or laparoscopic (*p* = 0.677) approach.

One patient that underwent transvaginal hysterectomy with bilateral oophorectomy received blood transfusion due to extreme bleeding caused by the previously undiagnosed Von Willebrand disease. However, it was not statistically different from 0 transfused patients in the laparoscopic group. Total rates of complications were quite similar in both groups, 1% for transvaginal and 3% for laparoscopic approach. Conversion of vaginal hysterectomy to laparotomy was necessary in one patient, to obtain bleeding control. In the laparoscopic group, pelvic hematoma was observed in one case and spontaneously resolved. None of the complications required reoperation. Consequently, there was no statistically significant difference between groups, comparing both complication and reoperation rates.

## 4. Discussion

Hysterectomy and bilateral oophorectomy are very important parts of female-to-male gender affirmation surgery, in both the esthetic and psychological respect [10]. Although laparoscopic, robotic-assisted, and vaginal hysterectomy have similar outcomes in females with benign gynecological disease, the vaginal route is currently associated with greater benefits, such as shorter operative times, lower infection rates, vaginal dehiscence, and conversion to laparotomy, as well as lower costs [11–14].

In female-to-male transgender populations, several specific aspects must be considered when planning the removal of female genitalia, including effects of testosterone treatment and the patient's high esthetic expectations. Long-term testosterone administration is associated with increased risks of intraoperative and postoperative bleeding and thromboembolic events, as well as possible gynecologic malignancy [15, 16]. On the other hand, its effect on the endometrium is still debated. While some data suggest that testosterone induces proliferative activity of the endometrium and hypertrophic myometrial changes, others report opposite effects [17, 18]. Lower uterine weight, compared to females without gender dysphoria, has also been reported [19]. It is therefore important to stop testosterone administration two weeks prior to surgery, in order to avoid excessive intraoperative bleeding. Some authors prefer laparoscopic hysterectomy in transsexuals due to better visualization of tissues and control of hemorrhage [3, 19, 20]. Gomes da Costa et al. reported 1 out of 23 transgender patients (4.3%) with significant postoperative bleeding, who underwent second-look laparoscopy and hemostasis [20]. O'Hanlan et al.'s study includes 41 transsexuals who underwent laparoscopic hysterectomy; one included a conversion to open laparotomy for observation of a large retroperitoneal hematoma (2.4%), while two (4.9%) required reoperation due to excessive bleeding [19]. Ott et al. performed conversion from laparoscopy to laparotomy in 1 out of 32 patients (3.1%), and none of the patients required reoperation [3]. There was no need for conversion of laparoscopic approach to laparotomy in our 32 cases, and reoperation rate was 0%. It is noteworthy that all previous studies evaluated hysterectomy performed in female-to-male transsexuals as a single procedure, or with vaginectomy and/or mastectomy. On the other hand, our group of 124 female-to-male transsexuals underwent simultaneous sex reassignment surgery, thus having a potentially increased risk of bleeding and associated complications. However, only 1 out of 124 patients required a blood transfusion, due to a coagulation disorder.

In case of our one-stage gender affirmation surgery, increased operative time could put the patient at a higher risk. This is why we prefer the transvaginal approach, as a significantly shorter procedure compared to the laparoscopic approach. Moreover, laparoscopic hysterectomy with bilateral oophorectomy requires additional, time-consuming activities, such as repositioning the patient and removing the equipment, in order to continue with sex reassignment surgery.

We did not experience any injury of vaginal mucosa during the vaginal approach that would compromise vaginal flaps for subsequent urethral lengthening, as a complication reported by other authors [20]. Kaiser et al., in one of the largest studies on vaginal hysterectomy in 103 female-to-male transsexuals, reported a complication rate of 5.4%, with mean duration of surgery of 52 minutes [21]. In 1 of our 92 patients (1%), it was necessary to convert the initial vaginal hysterectomy to abdominal hysterectomy due to excessive bleeding and hematoma formation. There were no other complications.

The potential shortcoming of this study is the disproportion in the number of patients in two groups; however, they are statistically comparable. In our study, both vaginal and laparoscopic hysterectomy have been proven to be safe, with minimal complication rates and without compromising simultaneous sex reassignment surgery (colpocleisis, urethral lengthening, metoidioplasty, and total phalloplasty). However, the laparoscopic approach is associated with a longer operative time and higher cost. It also requires three or four pelvic points of access in the abdominal wall, a visible trace of surgery, and a kind of stigma with potential psychological consequences in vulnerable patients. The scars in the anterior abdominal wall may also compromise abdominal phalloplasty, while possible injury of epigastric vessels with trocars may compromise microvascular anastomosis in total phalloplasty. These characteristics make transvaginal hysterectomy with bilateral salpingo-oophorectomy the optimal choice in transsexuals. The clear advantages of transvaginal approach are especially important in our setting of one-stage gender affirmation surgery, where a fast and the least invasive procedure, with minimal blood loss, is highly appreciated [5, 22]. Also, it is much easier and more comfortable to continue with sex reassignment surgery following transvaginal hysterectomy. Laparoscopic and robotic single-port access hysterectomy, less invasive than standard laparoscopy, may be a future alternative in transgender surgery, but current experiences are still quite limited [23, 24].

## 5. Conclusions

Comparisons of transvaginal and laparoscopic approach for hysterectomy with bilateral salpingo-oophorectomy remain relevant. Based on our experience, transvaginal approach as part of female-to-male gender affirmation surgery is safe, feasible, and valuable, bringing about numerous benefits. Laparoscopic hysterectomy presents a good alternative and could be recommended in selected cases.

## Figures and Tables

**Table 1:** Patients' characteristics in the two groups.

Route of hysterectomy	Number of patients (%)	Mean age (years, range)	Parity
Transvaginal	92 (74%)	28 (18–36)	2 (2%)
Laparoscopic	32 (26%)	32.5 (21–43)	0 (0%)

Total	124 (100%)	28.5 (18–43)	2 (1.6%)

**Table 2:** Comparison of results of transvaginal and laparoscopic hysterectomy with bilateral salpingo-oophorectomy.

Route of hysterectomy	Mean operative time (min)	Complications	Reoperation rate	Blood transfusion	Mean hospital stay
Transvaginal	51 (46–72)	1/92 (1%)^*∗*^	0/92 (0%)	1/92 (1%)	4 (3–6)^1^ 6 (5–8)^2^
Laparoscopic	76 (68–90)	1/32 (3%)^*∗∗*^	0/32 (0%)	0/32 (0%)	4 (3–6)^1^ 7 (6–8)^2^
*p*	<0.05	0.386	/	0.375	0.897

Total	57.5 (46–90)	2/124 (1.6%)	0/124 (0%)	1/124 (0.8%)	4 (3–6)^1^ 6 (5–8)^2^

^*∗*^Conversion of vaginal to abdominal approach. ^*∗∗*^Pelvic hematoma, spontaneously resolved. ^1^Metoidioplasty with urethral lengthening. ^2^Total phalloplasty with urethral lengthening.

**Table 3:** Duration of one-stage gender affirmation surgery in correlation with hysterectomy route.

Mean operative times (min, range)	Metoidioplasty	Total phalloplasty
Transvaginal hysterectomy	245 (215–325)	435 (390–550)
Laparoscopic hysterectomy	280 (240–375)	475 (410–590)
